# Evaluating the Risk Landscape of Hawaiian Monk Seal Exposure to *Toxoplasma gondii*

**DOI:** 10.1007/s10393-024-01678-7

**Published:** 2024-06-08

**Authors:** Stacie Robinson, Kim Falinski, Devin Johnson, Elizabeth VanWormer, Karen Shapiro, Angela Amlin, Michelle Barbieri

**Affiliations:** 1 NOAA, Hawaiian Monk Seal Research Program, Honolulu, USA; 2 UH, Water Resources Research Center, Honolulu, USA; 3 NOAA, Protected Species Division, Honolulu, USA; 4 University of Nebraska-Lincoln, Lincoln, USA; 5 University of California Davis, Davis, USA; 6 NOAA, Pacific Islands Regional Office, Honolulu, USA

**Keywords:** Hawaiian monk seal, Disease ecology, Hydrology, Risk model, Toxoplasmosis

## Abstract

Toxoplasmosis is a disease of primary concern for Hawaiian monk seals (*Neomonachus schauinslandi*), due to its apparently acute lethality and especially heavy impacts on breeding female seals. The disease-causing parasite, *Toxoplasma gondii*, depends on cats to complete its life cycle; thus, in order to understand how this pathogen infects marine mammals, it is essential to understand aspects of the terrestrial ecosystem and land-to-sea transport. In this study, we constructed a three-tiered model to assess risk of Hawaiian monk seal exposure to *T. gondii* oocysts: (1) oocyst contamination as a function of cat population characteristics; (2) land-to-sea transport of oocysts as a function of island hydrology, and (3) seal exposure as a function of habitat and space use. We were able to generate risk maps highlighting watersheds contributing the most to oocyst contamination of Hawaiian monk seal habitat. Further, the model showed that free-roaming cats most associated with humans (pets or strays often supplementally fed by people) were able to achieve high densities leading to high levels of oocyst contamination and elevated risk of *T. gondii* exposure.

**Supplementary Information:**

The online version contains supplementary material available at 10.1007/s10393-024-01678-7.

## Introduction

The risk of encountering infectious agents is typically highly variable across landscapes (Albery et al. 2022). Understanding the distribution of risk across the environment is a crucial step in strategizing disease response or prevention (Horan et al. 2005). For diseases that are widespread, easily transported, or exist in environmental reservoirs, disease management can seem insurmountable. However, much like systematic approaches aid conservation planners in identifying biodiversity hotspots for protection (Margules and Pressey 2000), using models to highlight areas of elevated risk and simulate disease response scenarios can provide critical insights to target limited resources where intervention can be most impactful. Toxoplasmosis (caused by the protozoal parasite *T. gondii* harbored by felids) is one such disease.

*Toxoplasma gondii* has a complex life cycle and is capable of infecting a wide range of warm-blooded species through waterborne and food web pathways (Aguirre et al. 2019). However, it can only complete its life cycle (sexual reproduction resulting in formation of oocysts) inside the intestine of felids (Hutchison et al. 1969; Dubey et al. 1970). Oocysts are shed in the 100 s of millions (Dubey 1995) and can persist and maintain infectivity for > 1 year in soil, freshwater, or saltwater, making them the critical parasite life stage that drives the environmental transmission (Dubey 1998; Miller et al. 1972; Lindsay and Dubey 2009; Shapiro et al. 2019a). Susceptible hosts can become infected if they encounter just a single one of these oocysts (Dubey et al. 1996; Hill and Dubey 2002). As oocysts have been spread widely by free-roaming felids, Toxoplasmosis has become one of the most common lethal food-borne diseases in humans (CDC 2023) and has threatened wildlife populations (Dubey et al. 2020; Lindsay and Dubey 2020).

**Figure 1:**
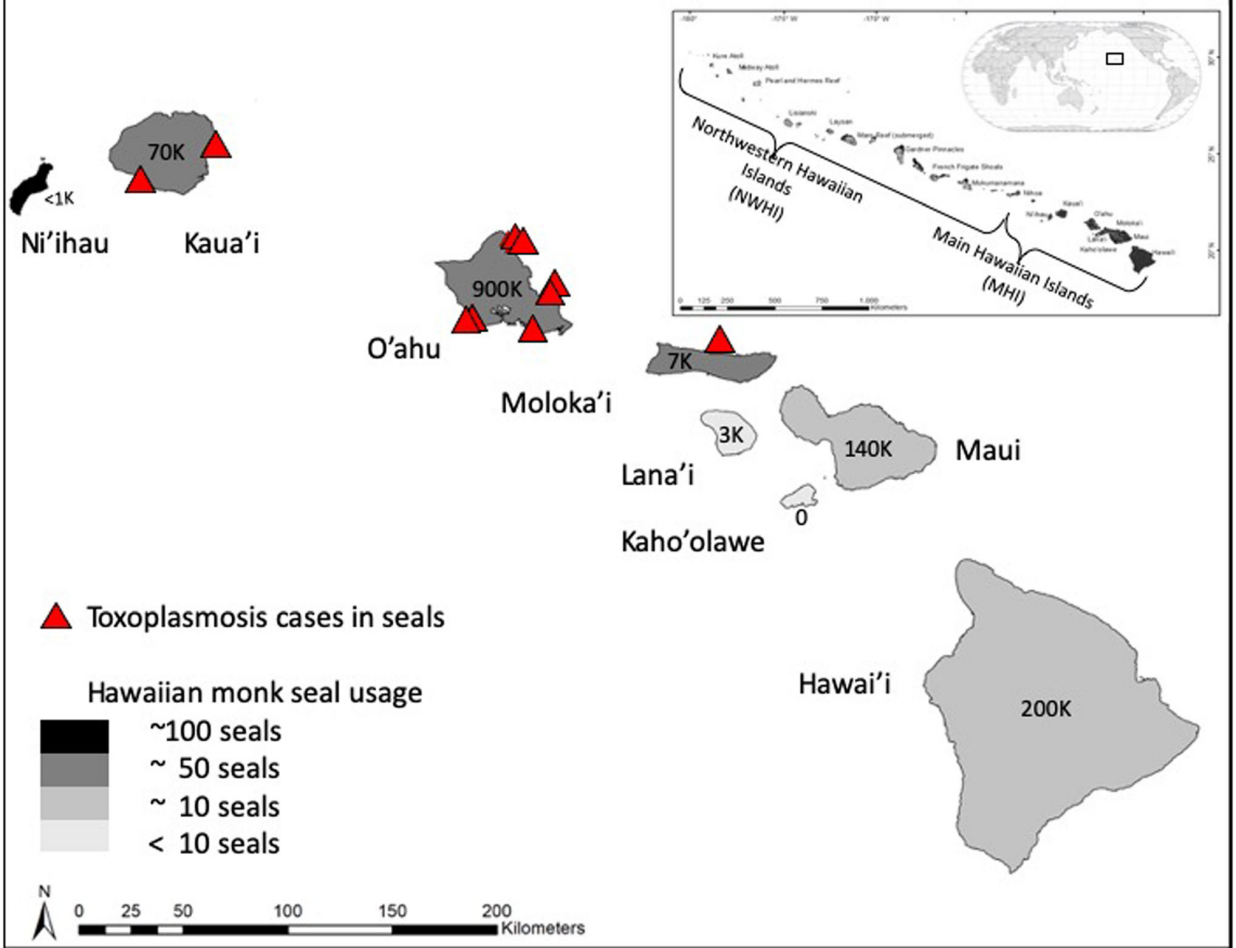
The map shows cases of toxoplasmosis detected in Hawaiian monk seals in the main Hawaiian Islands from 2001 to 2020. Along with cases (red triangles; defined per Barbieri et al. 2016), we provide context including the number of individual seals sighted annually on each island (Pacific Islands Fisheries Science Center 2022) and human population (US Census 2015). The inset shows the global context of the Hawaiian Archipelago, including Hawaiian monk seal habitat spanning both the main and Northwestern Hawaiian Islands (Color figure online).

Toxoplasmosis is the leading disease impacting endangered Hawaiian monk seals (*N. schauinslandi;* herein ‘seals’) in the human-inhabited main Hawaiian Islands (MHI; Fig. 1) (Harting et al. 2021). Although they constitute just more than 20% of the total seal population (~ 300 seals in the MHI and ~ 1100 in the Northwestern Hawaiian Islands estimated in 2020; Carretta et al. 2021), positive trends in the MHI have been one of the greatest signs of population rebound (Baker and Johanos 2004; Baker et al. 2016). But toxoplasmosis is dampening the species’ recovery by significantly reducing population growth potential in the MHI (Harting et al. 2021). Thus, understanding and mitigating the risk of Hawaiian monk seal exposure to this parasite is a critical goal of species recovery efforts (National Marine Fisheries Service 2007, 2021).

Seal deaths from toxoplasmosis have increased in prevalence in the MHI since the early 2000s (Barbieri et al. 2016), coincident with the timeframe of seals rebounding in the MHI, thus increasing opportunities for parasite exposure. Reports from other species demonstrate that *T. gondii* has long been established in the MHI. Early studies revealed that *T. gondii* was prevalent in cats on O'ahu (Wallace 1971). Infection has caused deaths in Hawaiian spinner dolphins (Migaki et al. 1990; Landrau-Giovannetti et al. 2022) and avian species (Work et al. 2000, 2002). The Hawaiian Island ecosystem is simpler than some continental systems in that Hawai'i has no native felids, leaving introduced domestic cats as the only contributors of oocyst pathogen pollution (Hess et al. 2007).

*Toxoplasma gondii* infection has been detected in numerous marine mammal species around the globe and in every ocean basin, demonstrating the ability of this terrestrial pathogen to infiltrate marine environments (Dubey et al. 2003; Gibson et al. 2011). Many marine mammals likely become infected by eating prey that have accumulated oocysts (Lindsay et al. 2001; Krusor et al. 2015; Massie et al. 2010) or through direct consumption of oocysts suspended in seawater (Conrad et al. 2005; Massie et al. 2010). Some predators may be infected by consuming *T. gondii* organisms encysted in tissues of infected prey (Jensen et al. 2010), but this is an unlikely route for seals which do not consume warm-blooded prey (Goodman-Lowe 1998; Cahoon et al. 2013). Additionally, animals including seals may acquire *T. gondii* through vertical transmission (Barbieri et al. 2016).

Research demonstrates that hydrological processes deliver oocysts from wide catchment areas into the marine environment (Simon et al. 2013). On the US west coast, cases of protozoal disease in sea otters have been linked to heavy rainfall and surface water runoff events (Shapiro et al. 2012, 2019b). Recent work has also demonstrated similar links between major runoff events and toxoplasmosis cases in Hawaiian monk seals (Robinson et al. 2023). While cat populations are thought to drive oocyst accumulation on the landscape (VanWormer et al. 2016), watershed features like slope, vegetative cover, soil types, or wetlands can impact water retention and soil capture.

**Figure 2:**
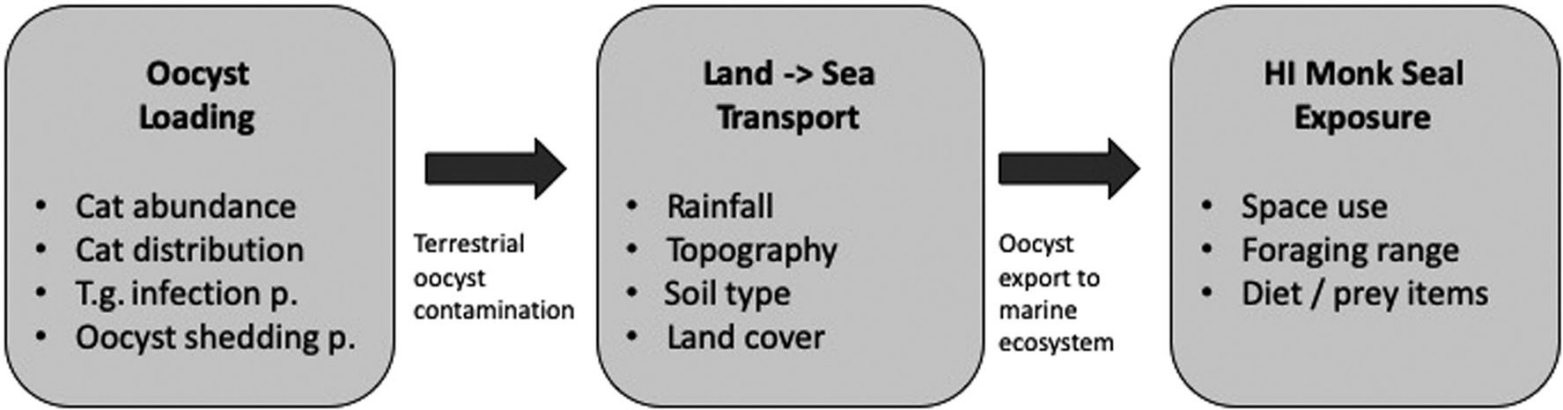
This flow chart shows components of the *T. gondii* risk model for Hawaiian monk seals. Boxes contain the key variables involved in each component of our risk evaluation. Text under arrows indicates how each component feeds into the next.

In order to better understand and manage disease risks, we take a multi-step modeling approach to examine ecological factors associated with *T. gondii* exposure for Hawaiian monk seals in the nearshore marine ecosystems of O'ahu, Hawai'i, USA (Fig. 2). This paper will address two research questions: (1) Are there spatial hotspots of toxoplasmosis risk for monk seals? (2) Do certain types of free-roaming cats contribute disproportionately to oocyst contamination? We will also develop a tool for scenario planning to aid disease risk mitigation efforts.

## Methods

### Oocyst Contamination: Cat Distribution and Oocyst Shedding

*Cat types and abundance*—Factors including wild prey availability, human care (feeding) and habitat characteristics influence the home ranges and diets of cats, shaping their density across the landscape and influencing *T. gondii* shedding (Zhu et al. 2021). The different contexts in which people see or interact with cats can influence their perceptions of cats and the acceptability of management actions (Lohr and Lepczyk 2014; Leong et al. 2020). Here, we considered outdoor cats of three types, capturing a gradient of human-association, ecological, and management situations (we closely followed the framework established in New Zealand’s Cat Management Strategy; New Zealand NCMSG 2016 and applied by Leong et al. 2020 while subdividing the umbrella category of free-ranging cats suggested by Lepczyk and Calver 2022). All domestic cats are the same species (*Felis catus*), all considered here are free roaming and have the potential to spread disease or prey upon native wildlife. The cat types do not constitute geographically or genetically distinct populations. We find these distinctions helpful in differentiating the relationships each cat type has with people, and thus differences in management actions and stakeholder groups involved with each. Additionally, we use landscape associations as another point of differentiation between cat types. For example, if a cat was located in a developed area, we would label it a stray cat that might have some association with humans/resources provided by humans. If a cat was located in undeveloped conservation lands, we would label it a wildland feral cat not interacting with or relying on humans. For the sake of simplicity, the model assumes more stark and static distinctions than would occur in the wild.

1. *Pet Cats* (household-associated pets) live with humans and are dependent on humans for their food, care and welfare. Here, we only consider the subset of pet cats that roam outdoors.
2. *Stray Cats* (whether independent or associated with colonies/feeding stations) do not live in households, but at least some of their needs are supplemented directly (colony feeding) or indirectly (dumpster foraging) by humans.
3. *Wildland Feral Cats* (wildland-associated strays) do not live around centers of human habitation and rely on hunting wild prey for sustenance. Feral cats exist at low densities in natural habitats (largely montane and forest on O'ahu) compared to strays in populated coastal communities (Smucker et al. 2000).

*Oocyst Shedding Prevalence—*We estimated oocyst deposition based on estimates compiled by Zhu et al. (2021) in a review of 127 studies finding that shedding prevalence in pet cats (whether kept solely indoors or indoor-outdoor) averaged 0.3–0.4%. Meanwhile, unowned cats (whether feeding solely on wild prey or supplemented by human feeding) had shedding prevalence averaging 3.2–4.1% (Zhu et al. 2021). We explored a range of plausible assumptions, including a high shedding prevalence of 4% for all cat types, and assuming a low shedding rate of 0.4% for pet cats. Because it is commonly asked whether stray cats in colonies might have lower prevalence due to human provisioning, we also ran stray cat models with reduced shedding prevalence of 2% (similar to findings of VanWormer et al. 2013). We estimated that each actively shedding cat sheds an average of 50,000,000 oocysts in a year (Afonso et al. 2010; Dabritz et al. 2007; VanWormer et al. 2016).

*Cat Distribution—*Landscape distribution scenarios were based on literature describing cat densities relative to landscape factors in Hawai'i or other areas (Cove et al. 2018; Goltz et al. 2008; Hess et al. 2009), and assumptions informed by input from seven professionals in Hawaiian conservation or animal welfare. As a first step, we determined the zone of suitable habitat for each cat type by considering their ecology and which land use/land cover (LULC) types each could occupy (LULC determined by NOAA’s Coast Change Analysis Program from aerial and satellite imagery at the 10 m resolution; Fig. SI1). This habitat suitability zone was used to exclude cats from highly improbable areas (such as open water and slopes > 40°). It was also used as a means of distinguishing cat types in the model. For instance, part of our definition of wildland feral cats was that they occupied undeveloped areas only; meanwhile, stray cats were defined by occurrence in developed areas.

In the most basic landscape distribution scenarios (LS Uniform), each cat type was distributed evenly across its habitat suitability zone (with the exception of pet cats, which we assume always have some amount of association with human households). In more complex landscape-weighted scenarios (LS Weighted), cats were distributed at 2 × or 3 × density in favored habitats (parameter details in Table SI2). We also constructed human household-associated scenarios (LS Household) in which portions of the cat population could be distributed based on household density (US Census 2015) with the remainder distributed throughout the habitat suitability zones.

### Land–Sea Transport: Hydrological Model

Oocyst transport has been documented in both surface and groundwater (Freppel et al. 2019; Shapiro et al. 2009; Vieira et al. 2015). We modeled oocyst export in an average year using the InVEST Nutrient Delivery Ratio (NDR) model which was created to run on large spatial scales with relatively simple input parameters. Further, the model accommodates both surface and subsurface flow (Sharp et al. 2015). Using inputs of slope, soil type, rainfall, erosivity, combined with the estimated retention of oocysts (or other modeled target) at each cell (10 m × 10 m), the model first calculates pathways of accumulation, then calculates the likelihood that a cell will deliver the load to the coast (see Fig. SI2). The final results are maps of oocyst contamination and export by watershed. The InVEST NDR model has previously been fitted to calculate statewide nitrogen export for Hawai'i (Falinski 2016). For retention rate and critical length parameters, we drew from extensive research parameterizing NDR models globally (Sharp et al. 2015) and followed O'ahu-specific work that had calibrated these factors based on sensitivity analysis (Falinski 2016; Hamel et al. 2017, parameters detailed in Table SI3).

### Oocyst Loading Model Sensitivity Analysis

All of the factors related to oocyst loading are imperfectly known for O'ahu, so our model represents a simulation based on data from literature, expert opinion, and assumptions. We assessed the impact that each of our assumptions could have on the model outcome by performing a sensitivity analysis using the one-at-a-time method described by Hamby (1994). We developed 36 model input scenarios based on altering the values for each variable across its reasonable range. We then assessed the degree to which changes in each input variable value altered model outcomes in terms of total oocyst export and spatial distribution of exports (e.g. which watershed showed elevated levels). The 36 scenarios covered combinations of 3 cat types, each with 3 abundance levels, up to 3 distributions, and up to 2 shedding prevalence levels (Table 1). Not every possible combination of parameters was considered realistic, so not all were run. For example, existing research would not support the idea that feral cats would have a low shedding prevalence, whereas outdoor pets or stray cats (particularly in colonies) might have a low shedding prevalence as some nutritional needs may be met by human feeding decreasing their exposure to *T. gondii* through wild prey (reviewed in Zhu et al. 2021). Oocyst loading from each of these scenarios was input into the hydrological model to estimate oocyst export from each O**'**ahu watershed. We base our proceeding risk analysis on the scenarios considered most likely to reflect baseline conditions: medium abundance, landscape-weighted distribution, low shedding prevalence for pet cats and high for all others (see Supplemental Information for all scenarios).

**Table 1:** Summary of Scenarios Simulating Varied Density, Distribution, and Oocyst Shedding of Cats on the O'ahu Landscape.

Scenario	Feral	Stray	Pet
A Low	1000	12,500	25,000
A Med	2500	25,000	50,000
A High	5000	50,000	100,000
SP High	4%	4%	4%
SP Low	NA	2%	0.4
LS Uniform	Even across HSZ	Even across HSZ	NA
LS Weighted	Favoring undeveloped/conservation land	Favoring roaded areas/trailheads	50% Even across HSZ, 50% with household density
LS Household	NA	50% associated with household density	100% associated with household density
No. Scenarios	3A * 1SP * 2LS = 6	3A * 2SP * 3LS = 18	3A * 2SP * 2LS = 12

We ran hydrologic models with a total of 36 scenarios varying abundance (A), *T. gondii* shedding prevalence (SP), and distribution on the landscape (LS) for each of four free-roaming cat types. HSZ = habitat suitability zone.

In addition to testing model sensitivity, it is useful to vary input variables according to potential outcomes of diverse management actions. For instance, what if a specific community were to decide to decrease their free-ranging cat population by a certain percentage? Or, what if a land management agency was weighing removing wildland feral cats from an upland preserve vs stray cats from a coastal harbor? We designed our model to be a useful tool to simulate outcomes of alternative management strategies. So, we integrated the model into an R shiny app to facilitate quick development of scenarios, generation of model inputs, and creation of oocyst distribution maps (user guide provided in SI).

### Hawaiian Monk Seal Exposure: Seal Space Use

Given the movement potential of seals (Cahoon 2011; Littnan et al. 2006; Wilson et al. 2017), stranding locations do not represent exact locations of *T. gondii* exposure; however, the aggressive and severe nature of toxoplasmosis in seals suggests that recent exposure in the region of stranding is likely (Barbieri et al. 2016). Here, we calculated seal utilization distributions based on satellite telemetry data collected from 91 instruments deployed on 70 seals in the MHI between 2007 and 2019, representing 18% of seals known to inhabit the MHI within this time period (Cahoon 2011; Littnan et al. 2006; Wilson et al. 2017; Robinson and Littnan 2020; Pacific Islands Fisheries Science Center 2022). Each deployment had at least 15 days of data (mean = 94.52, max = 229). Using the argosfilter package (Freitas 2015) for the R statistical environment (R Core Team 2013), we filtered out locations likely to be erroneous based on unrealistic speeds or turning angles. We then used the R package crawl (Johnson and London 2018; Johnson et al. 2008) to simulate locations at 15 min time intervals from the Bayesian posterior distribution of estimated tracks (Johnson et al. 2011). Then, we calculated utilization distributions (UD) on a 1 km grid for each deployment and track, measuring the proportion of deployment time that each seal spent in each grid cell. Using the imputation approach (McClintock 2017; Scharf et al. 2017) we were able to normalize all deployments to have equally dense location intervals while accounting for location uncertainty caused by in-filling locations. We averaged the proportions of each seal’s space use in each grid cell to construct a compiled UD representing generalized space use for seals in the main Hawaiian Islands.

### Hawaiian Monk Seal Exposure: Risk Zones

Our analysis showed the vast majority of seal space use occurred at depths less than 100 m. Thus, we used the 100 m isobath as a bound for the nearshore area where seals might be most exposed to oocysts entering the marine environment with terrestrial runoff. We extended watershed boundaries out to the 100 m isobath to define the exposure risk zones (ERZs) where monk seals would be exposed to the oocyst export from each watershed estimated by the hydrological model. Some were combined or subdivided to make more evenly sized zones.

To evaluate overall risk of *T. gondii* exposure, we considered both seal usage and oocyst export into each ERZ. First, we summed seal space utilization within each ERZ and coded each according to five quantiles of seal usage. Next, we added oocyst exports from all cat types (based on the most likely scenario) to summarize the total exported to the ERZ from the adjacent watershed and coded each according to five quantiles of total oocyst export. Finally, we multiplied the seal usage code by the oocyst export code to provide the overall exposure risk in each ERZ (color coded in Fig. 5). Areas with elevated risk scores could be considered hotspots. We used Spearman’s rank correlation test to evaluate the correlation between risk zone ranking and occurrence of toxoplasmosis cases in monk seals (performed using cor.test routine in R; R Core Team 2013).

## Results

### Oocyst Contamination: Cat Distribution and Oocyst Shedding

**Figure 3:**
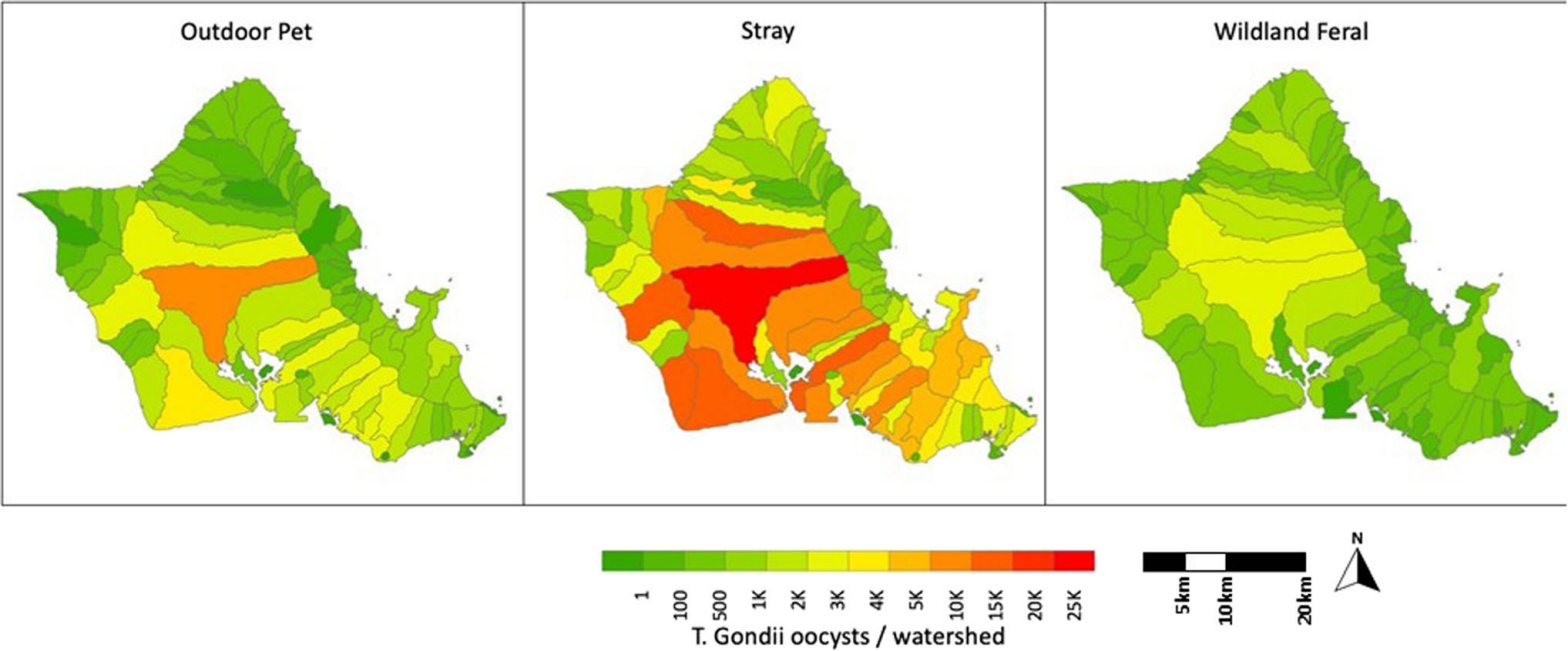
Maps show spatial distribution of annual *T. gondii* oocyst contamination across O'ahu contributed by three types of outdoor cats. For each map, we use the ‘most likely’ scenario for the given cat type: medium abundance, landscape-weighted distribution, and oocyst shedding prevalence derived from literature (0.4% for pet cats, 4% for wildland feral and stray cats; Zhu et al. 2021).

The broad scale landscape distribution scenarios (habitat suitability zones) applied to each cat type had a strong influence on the distributions of oocyst contamination. While both pet and stray cats occupied overlapping areas, based on our distribution rules, wildland feral cats occurred less densely and occupied a nearly reciprocal area of the island (Fig. 3). Meanwhile, the finer distinctions between landscape weighting scenarios had a limited impact on oocyst contamination patterns (Fig. SI3 for all scenarios).

### Land–Sea Transport: Hydrological Model

**Figure 4:**
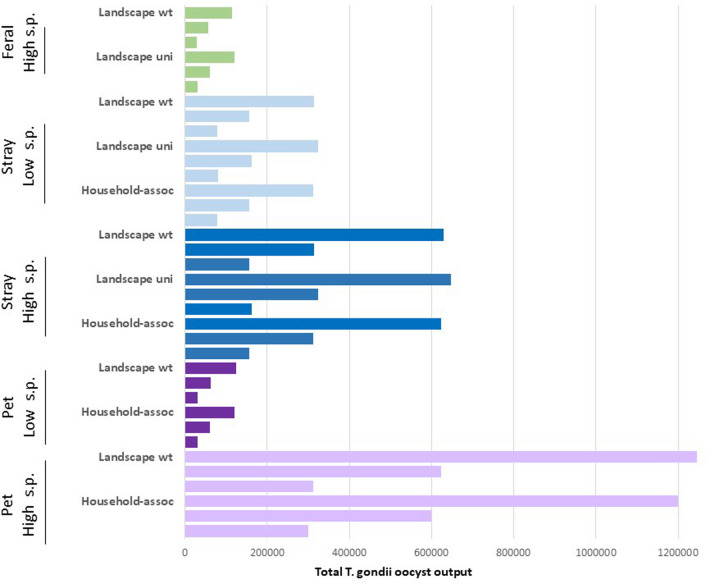
Total annual oocyst outflow (colored bars) from O'ahu according to hydrological models under scenarios varying abundance, distribution, and shedding prevalence of each cat type. For each scenario, bars are arranged along the by High, Med, Low abundance levels (top to bottom on *Y* axis). LS indicates landscape scenarios. Light blue and light purple bars represent shedding prevalence (SP) scenarios considered less likely according to literature. The *X* axis shows the model-estimated total oocysts exported to coastal waters of O'ahu (Color figure online).

*Sensitivity Analysis—*Oocyst exports increased linearly as cat abundance increased. Oocyst exports also varied linearly with shedding prevalence, which, given the ten-fold range suggested in the literature, made this a highly influential parameter. If shedding prevalence was assumed to be equal across all cat types, oocyst export from pet cats would dwarf that of all other cat types (faded bars in Fig. 4). However, assuming shedding rates consistent with Zhu et al. (2021), stray cats became the greatest contributors to total export (dark bars in Fig. 4).

Controlling for a specified abundance level and shedding prevalence, the landscape-weighted distribution scenarios had minimal impact on total oocyst export (Fig. 4). Across landscape distribution scenarios, the vast majority of oocysts deposited across O'ahu were retained on the landscape with only ~ 0.06% being exported to coastal waters (varying from 0.057% for low-density wildland feral cats in vegetated uplands to 0.065% for high-density stray cats in developed coastal areas). Given the high levels of contamination, even this small percentage resulted in 10,000 s to 1,000,000 s of oocysts reaching coastal waters around O'ahu in each annual model cycle (varying by scenario, Table SI2).

### Hawaiian Monk Seal Exposure: Seal Space Use

Seals used all nearshore (coastline to 100 m isobath, Fig. SI7) regions of the O'ahu coast, but usage was higher on the leeward side of the island, with additional clusters of heavy space use at the northeast and southeast points. The central north shore and most of the windward side were less lightly used by telemetered seals (Fig. SI7).

### Hawaiian Monk Seal Exposure: Risk Zones

**Figure 5:**
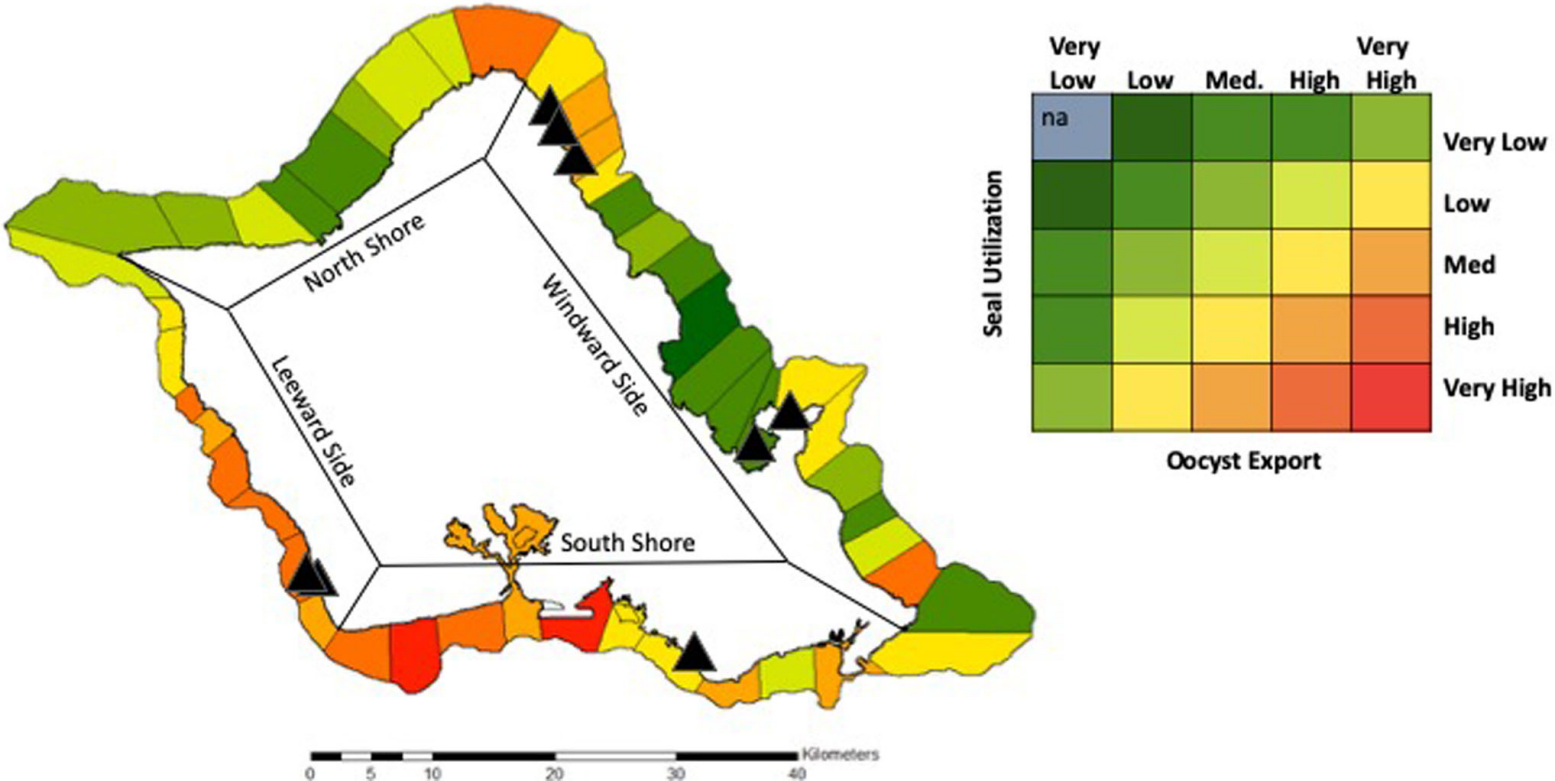
Exposure risk zones are coded based on area utilization by Hawaiian monk seals and total annual *T. gondii* oocyst export from adjacent watersheds. Oocyst export was calculated using hydrological models combining exports from all four cat types (based on ‘most likely’ scenario for each cat type and summed by watershed). Seal habitat utilization was based on compiled telemetry data. Black triangles show detected seal toxoplasmosis cases for context. Levels Very Low–Very High represent quantiles of the respective metrics (Color figure online).

While risk of oocyst exposure varied across O'ahu, all zones presented some level of risk (no zones were classified in lowest quantile for both seal usage and oocyst export; Fig. 5). Our model suggests that the greatest risk of *T. gondii* exposure for seals was in the southwestern quadrant of O'ahu (southern leeward side and western south shore) where both oocyst export and seal use fell in the highest quantile (Fig. 5). Northeastern and southeastern points of O'ahu showed moderate risk given medium to high oocyst export and medium to high seal usage. Most of the north shore and central windward side showed lower risk given low seal use and low to medium oocyst export. Correlation tests showed that numbers of cases were positively, though not significantly, correlated with risk zone scores (rho = 0.12, *p* = 0.42).

## Discussion

### Potential Hotspots of *T. gondii* Exposure

By considering terrestrial oocyst contamination, land-to-sea hydrologic flow, and seals’ coastal space use, we were able to identify hotspots of elevated risk for Hawaiian monk seal exposure to *T. gondii.* Stranding locations of seals with toxoplasmosis showed some alignment with the risk zones identified by the model (Fig. 5), providing insights into the more influential risk factors for exposure. General spatial patterns were consistent across outputs from all oocyst export scenarios (most likely scenario in Fig. 5, all others Figs. SI4–SI6), with the south shore and leeward side of the island having the greatest expected oocyst contamination. These represent the areas of greatest urban development on O'ahu. In such areas, the threat of highly concentrated human-associated cats also offers great opportunity to decrease localized risk through management of human actions.

Two areas on O'ahu have become focal points for multiple toxoplasmosis cases (Barbieri et al. 2016). One area was the southern leeward side where the model estimated some of the highest risk levels, indicating good alignment between model-predicted risk and observed seal stranding locations. The northern windward side has accumulated 3 cases, and here modeled risk estimates were moderately elevated compared to surrounding zones. The lack of statistically significant correlation between toxoplasmosis cases and risk scores could indicate: (1) the limited number of seals and toxoplasmosis cases limits detection of statistical patterns, (2) based on seal movement capabilities or ocean water movement and mixing stranding locations may be a poor representation of locations of oocyst exposure, (3) the high infectivity of oocysts could mean that risk operates on a threshold where, above moderate contamination levels, added oocysts do not linearly increase risk. This last possibility would comport experimental findings in other species that have shown that a single oocyst is sufficient to infect susceptible hosts (Dubey et al. 1996; Hill and Dubey 2002).

Dietary preferences can influence risk of disease exposure in wildlife. For instance, in Southern sea otters, snail specialists were more likely to be infected with *T. gondii* (Johnson et al. 2009). However, difficulty observing feeding (Parrish et al. 2008) and the wide variety within seal diets (Iverson et al. 2011; Longenecker 2010; Cahoon et al. 2013) have inhibited discerning dietary associations. In the MHI, studies indicate favoritism for shallow near-shore areas, with prey searching and capture activity concentrated around 25.3 ± 16.2 m (Wilson et al. 2017). So, while it may be difficult to say which food items pose the greatest exposure risk to seals, we can surmise that the nearshore zone, where seals catch and consume most of their prey, is the most likely area of exposure. And given the proximity to freshwater outflows, this is unfortunately the zone most likely to accumulate oocysts from terrestrial runoff. This increases risk of encountering oocysts whether through food items, sediment, or in the water column.

### The Role of the Landscape

Our model results predicted that oocysts were transported from land to sea at similar proportions across all landscape-weighted distribution scenarios. While wetlands and estuaries have potential to capture and retain oocysts (Hogan et al. 2013; Simon et al. 2013; Shapiro et al. 2010), we did not see substantially lower export from watersheds with wetlands in the model results. But, as O'ahu is characterized by short steep watersheds with small alluvial plains and high-intensity seasonal rainfall, these systems may have been overwhelmed by the volume oocyst contamination running off from heavily populated coastal regions. While not a key strategy for mitigating toxoplasmosis risks for marine life, restoring coastal wetlands has numerous benefits for the reef ecosystem of Hawai'i (Fabricius 2005; Wolanski et al. 2009). However, without also addressing free-roaming cat overpopulation, oocyst accumulation in estuarine environments could pose threats to wetland wildlife.

### The Role of the Cats

Cat abundance and shedding prevalence were the most influential factors in seal exposure risk to *T. gondii*. We found that human-associated cats contributed the most to oocyst contamination on the island. Varied assumptions about shedding rates for pet and stray cats influenced which might be the highest risk group. Additional data about shedding prevalence in the O'ahu population could help distinguish risk levels. However, at any level of toxoplasmosis, both cat types occur in high numbers and present substantial risk. Managing human actions regarding cats (allowing pets outside, maintaining stray colonies) to reduce the numbers of these human-associated cats roaming the landscape will be key in reducing seal exposure risk.

We modeled wildland feral cats at even higher densities than suggested by literature (Cove et al. 2018; Goltz et al. 2008; Hess et al. 2009); yet still, wildland feral cats had the least impact on overall oocyst export. It is unlikely that wildland feral cats could reach densities that result in oocyst contribution at the level of more urban cat types (it would require 23 wildland feral cats per km^2^ of habitat to reach the estimated number of stray cats). Even if not the top contributors to toxoplasmosis in marine life, this group of cats is likely the most impactful on sensitive avian species in Hawai'i through predation and upland disease spread, and thus remains a high conservation priority (Hess et al. 2009; Smucker et al. 2000; Raine et al. 2020).

### Limitations and Future Directions

*Nature of T. gondii—T. gondii* oocysts exhibit properties such as small size, hydrophilic nature and negative charge that facilitate their transport in freshwater (Shapiro et al. 2009). While these properties suggest that oocysts would be less apt to settle out of freshwater runoff than sediment particles (with greater mass and less/no charge), we acknowledge that treating oocysts as dissolved nutrients in the InVEST NDR model represents an oversimplification that may overlook some complexities in the system. As another simplification, our model assumed that 90% of oocyst transport occurred through surface hydrology; however, the porous geology of volcanic islands may facilitate groundwater transport as well (Bishop et al. 2015). Yet, even if the absolute number of oocysts reaching the coasts is mis-estimated, the model remains an informative tool to examine the relative contribution of cat types and specific watersheds to coastal oocyst contamination. These relative measures and maps can help direct risk mitigation strategies, particularly given limited resources. Models simulating relative outcomes of different scenarios (such as population viability models) have proven valuable in conservation planning (Fantle-Lepczyk et al. 2018).

*Shedding Prevalence*—The shedding prevalence parameters used in our model were derived from literature and represent a wide range of plausible shedding prevalence (varying by region and cat type; Zhu et al. 2021). This input could be refined with specific data on shedding of different cat types on O'ahu or through multiple imputations to generate a distribution. Further, better understanding the diversity of *T. gondii* genotypes circulating in the island ecosystem could refine our understanding of risk levels (Shapiro et al. 2019a, b; Xiao and Yolken 2015). If actual shedding rates were found to differ widely from our assumptions, this effect would be most realized in shifts in the relative contribution of oocysts from colony versus pet cats. The relative risk map is likely to remain consistent regardless of changes in the actual numbers of oocysts shed.

*Cat Population Data*—In our model, we had the most direct data about pet cat populations (Ward Research Inc. 2018), whereas the other cat types were estimated based on literature and habitat available. Improved data on both the abundance and landscape distribution of un-owned cat types could refine predictions related to *T. gondii* risk and allow better evaluation of management efforts. At the time of this research, population assessments of free-roaming cats have been a point of conversations, proposed legislation, and pilot studies on O'ahu. Well-designed cat counts have provided valuable data in other urban areas (Cove et al. 2018) and could provide more accurate resolution of oocyst contamination, serving as a baseline against which to measure effects of cat management actions.

*Time Frame—*We used an annual model which accounts for both oocyst contamination and rainfall as annual averages. This means that we may miss the effects such as large flushes of oocysts with abnormal rainfall events, or higher levels of accumulation with prolonged droughts. Additionally, the annual model does not account for the potential accumulation of oocysts across years which is likely given the longevity of oocysts in the environment (Dubey 1998). But here again, we can rely on the utility of relative measures. Those watersheds accumulating the most oocyst contamination in the annual model are likely the areas to accumulate the most year after year.

## Conclusions and Implications

Even with the above data gaps, sufficient information is available to characterize patterns of toxoplasmosis risk and we believe our model provides actionable information to natural resource managers and community stakeholders, as well as a valuable framework for thorough consideration of wildlife disease in the face of uncertainties. A variety of managers and stakeholders can apply the model to their own scenarios using the R shiny app available at https://connect.fisheries.noaa.gov/content/d1069bd2-c505-42d8-bee5-b04cbad6e5ea/.

Our model demonstrated that under a wide range of realistic conditions, human-associated cats (whether pet or colony) pose the greatest risk of *T. gondii* contamination throughout the ecosystems of O'ahu. Our model also showed that elevated risk of *T. gondii* pathogen pollution was widespread across the island, not isolated to a few hotspots. While the wide distribution of risk may appear daunting, the importance of human-associated cats highlights the power that people have to make positive impacts in reducing risk of toxoplasmosis. Careful management and reduction of the free-roaming cat population will be an essential step in reducing the risk of toxoplasmosis for other species.

As the seal population continues to recolonize the part of its range that overlaps with dense human populations (MHI), it is reasonable to expect increases in the risk of toxoplasmosis*.* This represents a critical challenge in Hawaiian monk seal conservation, threatening the growth of the MHI population which is a crucial component of the species’ recovery (National Marine Fisheries Service 2007, 2015, 2021,). While management efforts might start with one or more high-risk watersheds, landscape level strategies will be important in managing cat populations and mitigating this disease threat to wildlife in Hawai'i.

## Supplementary Information

Below is the link to the electronic supplementary material.

Supplementary file1 (DOCX 759 KB)

Supplementary file2 (XLSX 169 KB)
