# Supplementary material for: Evaluating the Risk Landscape of Hawaiian Monk Seal Exposure to Toxoplasma gondii
Source: Ecohealth. 2024 Jun 8;21(2-4):141–54. doi: 10.1007/s10393-024-01678-7 (PMC11649717; doi:10.1007/s10393-024-01678-7)
Supplement: Supplementary file 1 — Supplementary file1 (DOCX 759 KB) [file 10393_2024_1678_MOESM1_ESM.docx]

**SUPPLEMENTAL INFORMATION**

**Evaluating the risk landscape of Hawaiian monk seal exposure to Toxoplasma gondii**

Robinson et al.

### Supplemental Methods - Details for cat distribution scenarios

Criteria applied to all cat types

*Open water*

Because cats are terrestrial, we excluded cats of all types from areas classified as Open Water.

*Slope*

While high slope may not prohibit utilization by cats, resident density would likely be minimal. Initial model development showed that there were concerns about areas of high slope where runoff was predicted to occur disproportionately, and the delivery ratio was unrealistically high.  These areas were steep cliffs in the very highest parts of the Ko'olau Mountains. Because the slope calculation may go towards infinity in the accumulation step of the model, and, given the minimal expectation of cat utilization of steep cliffs, model developers we remove areas with slope greater than 40^o^.  The area of O'ahu that has slope higher than 40 degrees accounts for 8.9% of the total area, mostly in upper reaches of conservation land use areas.

Companion cats

*Available data*

Telephone surveys of residents indicate that outdoor pet cats on O'ahu number upwards of 50,000 animals (19% of ~316,000 O'ahu households own an average of 2.2 cats, ~50% allowed to roam outside; Ward Research 2012, 2018, US Census 2015). The Ward Research Survey from 2018 was conducted by a professional market research firm, under contract to the Hawaiian Humane Society. In 2018 there were 403 respondents. 19% of Hawai'i households owned a pet cat. Of cat owners 58% (O'ahu) / 46% (other islands) owned a single cat, while 42% (O'ahu) / 54% (other islands) owned multiple cats. According to the 2012 Ward Research Survey, A majority of cat owners allowed their pets to roam freely outside (52% on O'ahu / 71% other islands). This question was not repeated in 2018.

*Distribution scenarios*

By their definition, companion cats must be associated with human households, and thus developed areas. In our model, we included companion cats in land use areas zoned Urban (O'ahu has no Rural zoned areas), or Agricultural with development. They were excluded from land zoned Agricultural without development or Conservation land (where housing is generally not permitted, indicating any cat present would best fit in other categories). Within developed areas, pet cats might be equally likely across all household types, but pet cats are less likely to roam outdoors in high density housing buildings such as multi-story apartment buildings where it would be difficult for cats to transit in/outdoors. Thus, we removed households in buildings containing 10 or more units before computing household density (based on US Census data). Within each census tract, we constrained the households to developed land use types. Cats density was then mapped in association with households.

Stray cats

*Available data - Colonies*

Cat colonies are common on O'ahu with 4% of telephone survey respondents answering that they fed >5 cats they did not own, which could be taken as indication of colony caretaking. ( Ward Research 2018). However, since most colonies are fed by numerous people, and some feeders manage numerous colonies, it is difficult to translate these percentages into island-wide colony cat populations. A previous study on O'ahu estimated over 16,700 colony cats based on a caretaker registry ( Lohr et al. 2013). Based on the continued input of cats to colonies ( Ward Research 2012) we rounded up to use 20,000 colony cats as our medium population estimate.

*Distribution scenarios*

In our model, we included colony cats in land use areas zoned Urban (O'ahu has no Rural zoned areas), Agricultural with development, or Conservation land with development (since unlike companion cats, colonies are likely to be maintained in parks, campgrounds, or trail areas within Conservation land). They were excluded only from undeveloped lands (zoned either Agricultural or Conservation) where lack of infrastructure was likely to indicate limited access to colony caretakers.

*Available data – non-colony associated*

A study in the Florida Keys (sharing some similarities as islands with mild climates) found ~4 stray cats/km^2^ in suburban areas ( Cove et al. 2018). If we use a similar estimate, developed areas of O'ahu (570 km) could support nearly 2,500 stray cats. However, this number seemed low in comparison with anecdotal reports (pers. comm. Hawaiian Humane Society, 2020), so we used 2,500 stray cats as our low population estimate.

*Distribution scenarios*

In our model, we included stray cats in land use areas zoned Urban (O'ahu has no Rural zoned areas) or Agricultural with development. They were excluded from land zoned Agricultural without development or Conservation land (with or without development). We acknowledge difficulty separating these strays from cats that might be free-roaming companion cats or colony cats (for example in responses in the Ward Research surveys). It is important to acknowledge that, while we will model these populations separately, it would require careful survey designs to get accurate counts of the cats in these categories that may spatially overlap.

Wildland feral cats

*Available data*

Studies on Mauna Kea (on Hawai'i Island) found feral cats in rugged montane landscapes occurred at relatively low density and exhibited some of the larger home ranges reported for feral cats (7.72 km^2^ for females, 14.18 km^2^ for males; Goltz et al. 2008, Hess et al. 2009). Similarly, the Florida Keys study showed cats at about 4X lower density in remote areas (Cove et al. 2018). Thus, while we do not have specific estimates of feral cat abundance on O'ahu, we assumed low densities in the typically rugged areas beyond human developments. If, again, we use estimates similar to the Florida Keys population (1 cat / km), undeveloped areas of O'ahu (882 km) would support nearly 1,000 feral cats. However, this number also seemed low in comparison with anecdotal reports (pers. comm. Hawaiian Humane Society, 2020), so we used 1,000 feral cats as our low population estimate.

*Distribution scenarios*

In our model, we included feral cats in land use areas zoned Agricultural without development or Conservation land (with or without development). For the purposes of this modeling exercise, cats in developed areas not associated with colonies or households will be counted in the Stray category. Given the adaptive and widespread nature of feral cats, and the lack of habitat-specific abundance data on O'ahu, we constructed a variety of scenarios to test the impact of different distributions.

**Supplemental Methods – Model details**

The NDR model inputs include accumulation rates for each cell (calculated from the scenarios above) and a retention rate based on the LULC class. Additional parameters for the model include the critical length, defined as the distance from a given cell after which it is assumed that a patch of LULC retains the constituent at its maximum capacity (see Table SI3), and the proportion of constituent moved in subsurface flows. Following the work of Kwong et al. (2002), we assumed that most (90%) of the constituent load was exported in the surface runoff rather than through subsurface flow (Kwong et al. 2002). The subsurface critical length was set to 50 m and the maximum retention efficiency at 0.99 (unitless ratio, dissolved nutrient retained by a given land use class). Parameters for all scenarios and LULC classes in Table SI3. For the threshold flow accumulation value (1250 cells) and Borselli k factor values (2, unitless constant for soil erosivity), we followed Hawai'i-specific work that had calibrated these factors based on sensitivity analysis (Hamel et al. 2017, Falinski 2016,).

**Supplemental Methods Graphics**

**Figure SI1: Cats were distributed across the O**'**ahu landscape based on land use / land cover classifications and habitat occupation rules for each cat type.** The table provides the legend for LULC classes and indicates whether a given cat type may occupy habitat of that class (and the preference weight under each scenario).

Abbreviations: Prev = Pervalence, Dist = Distribution, HH = Household, Cons = Conservation, Ag = Agriculture, Dev = Developed, Un = Undeveloped, Rds = Roads buffered by 50m.


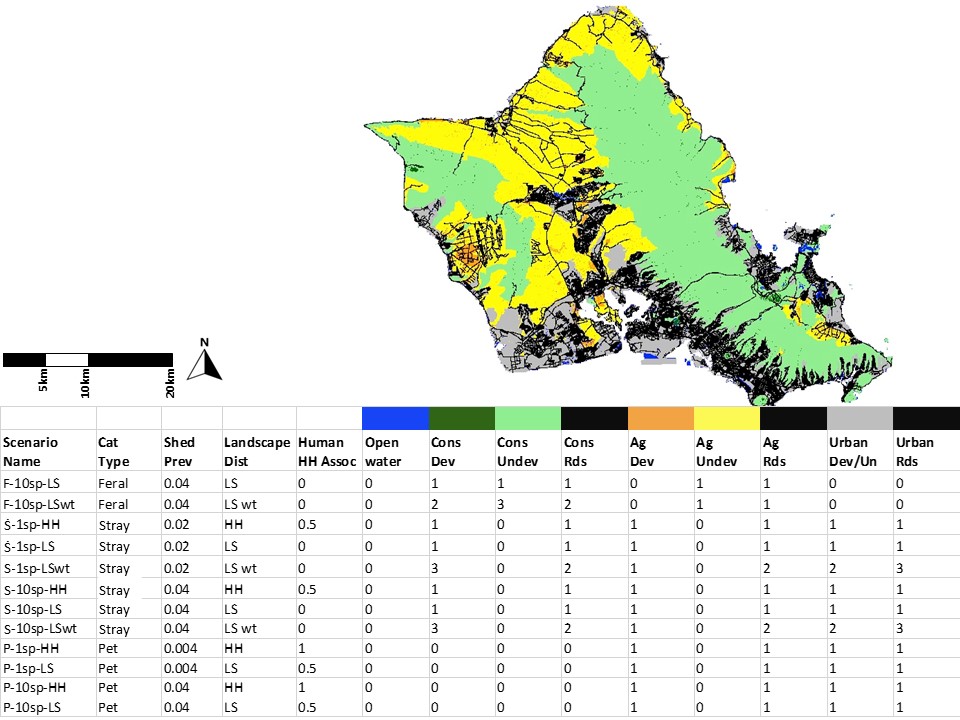


**Figure SI2: Conceptual diagram of the InVEST NDR model with delivery ratio calculated for the Honolulu metro area.** The unit of export is the same as the unit of the loading, which in this case is the total number of oocysts. This map illustrates the general hydrological model output with variation in nutrient delivery ratio based solely on hydrological characteristics of the O'ahu landscape (before accounting for oocyst loading).


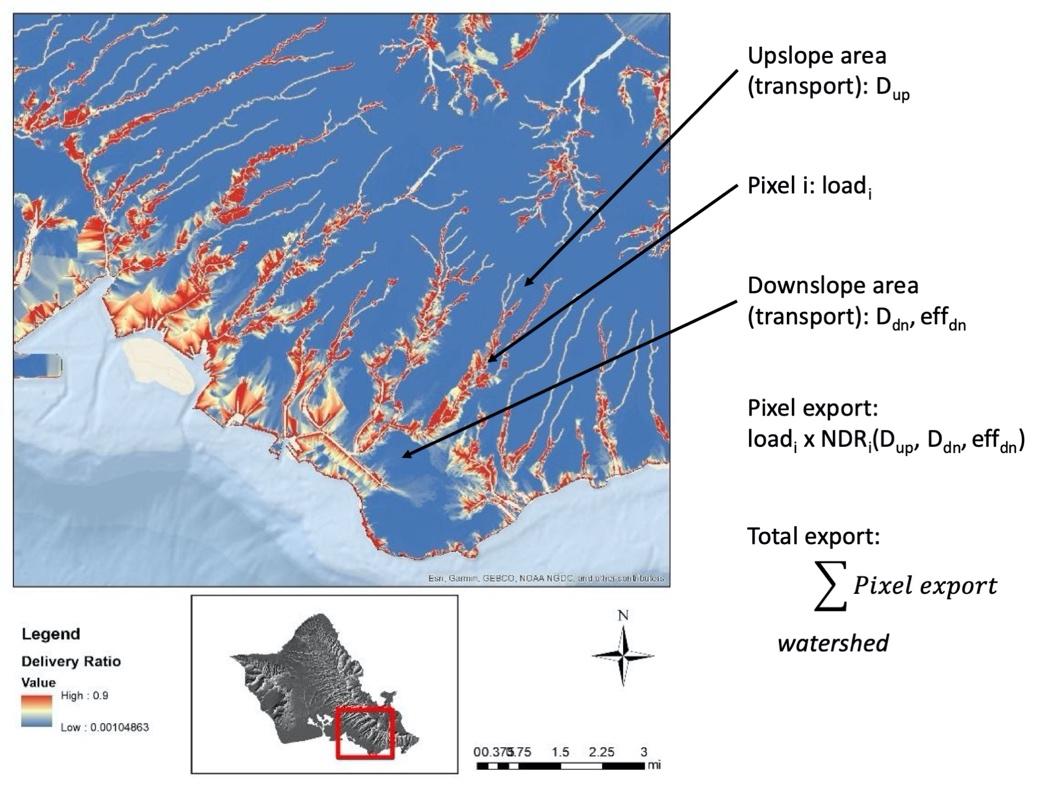
Model terms:

D_up_ – the amount of the modeled contaminant delivered from up slope cells to cell_i_.

D_dn_ – the amount of the modeled contaminant delivered from cell_i_ to down slope cells.

eff_dn_ – the efficiency of transport out of cell_i_ (based on slope, soil, landcover).

load_i_ – the amount of the modeled contaminant contained in cell_i_.

NDR_i_ – the Nutrient Delivery Ration which is a function of the other parameters listed.

**Supplemental Results Graphics**

Oocyst Loading: Cat distribution and oocyst shedding

### Figure SI3: Oocyst loading maps for all landscape distribution scenarios (medium cat abundance and most likely shedding prevalence assumed for all scenarios, annual model).


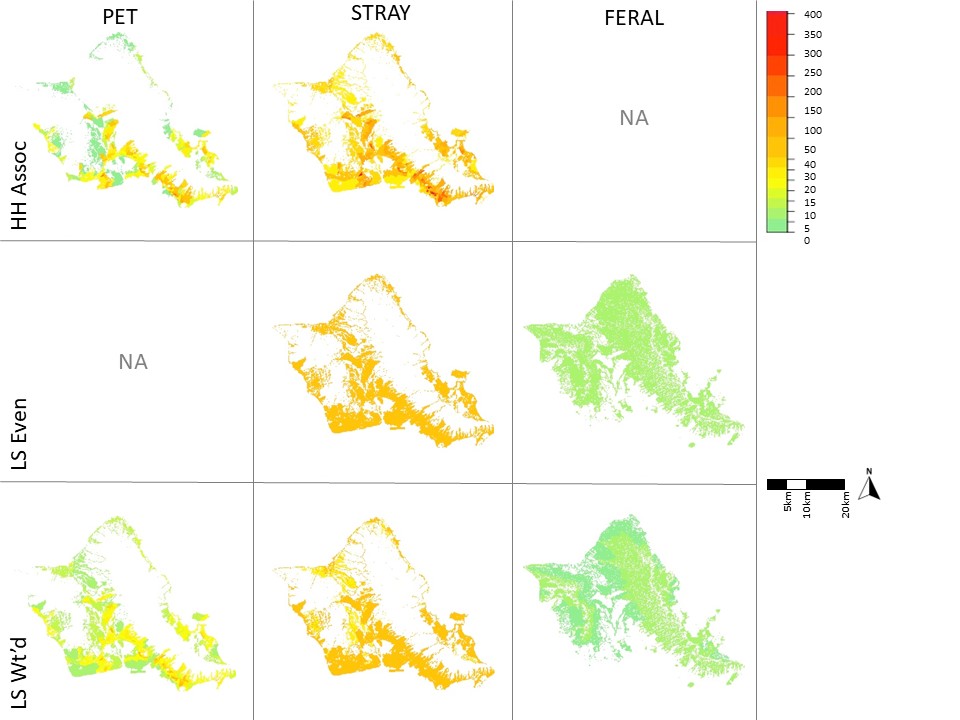


Land-sea transport: Hydrological model outputs

**Figure SI4: Oocyst export summarized by watershed for all landscape and shedding prevalence scenarios for pet cats (medium abundance, annual model).**


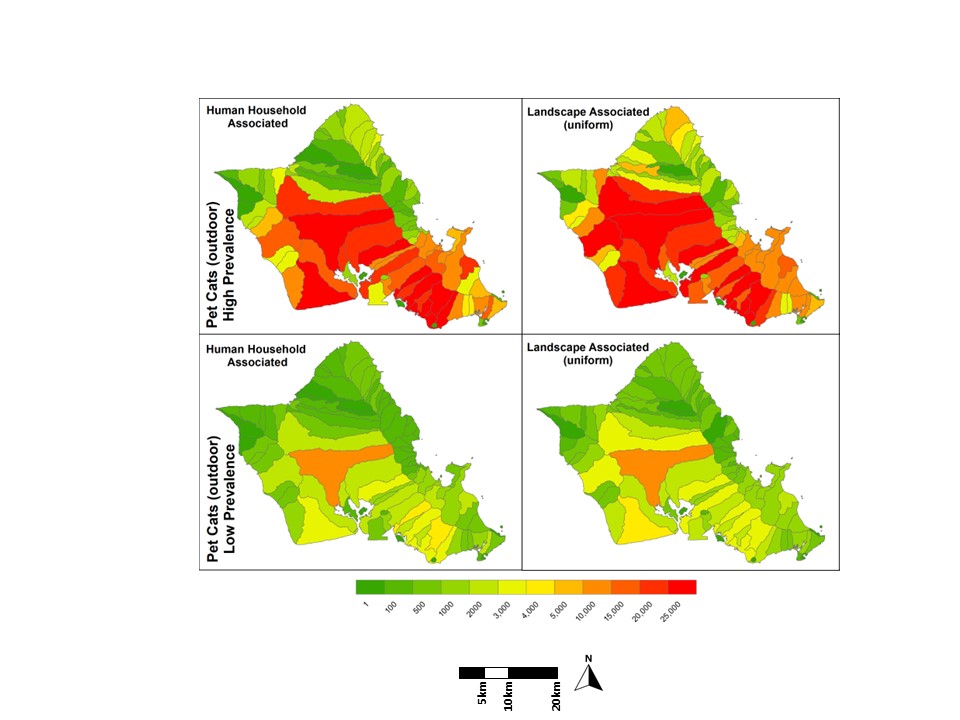


### Figure SI5: Oocyst export summarized by watershed for all landscape and shedding prevalence scenarios for colony cats (medium abundance, annual model).


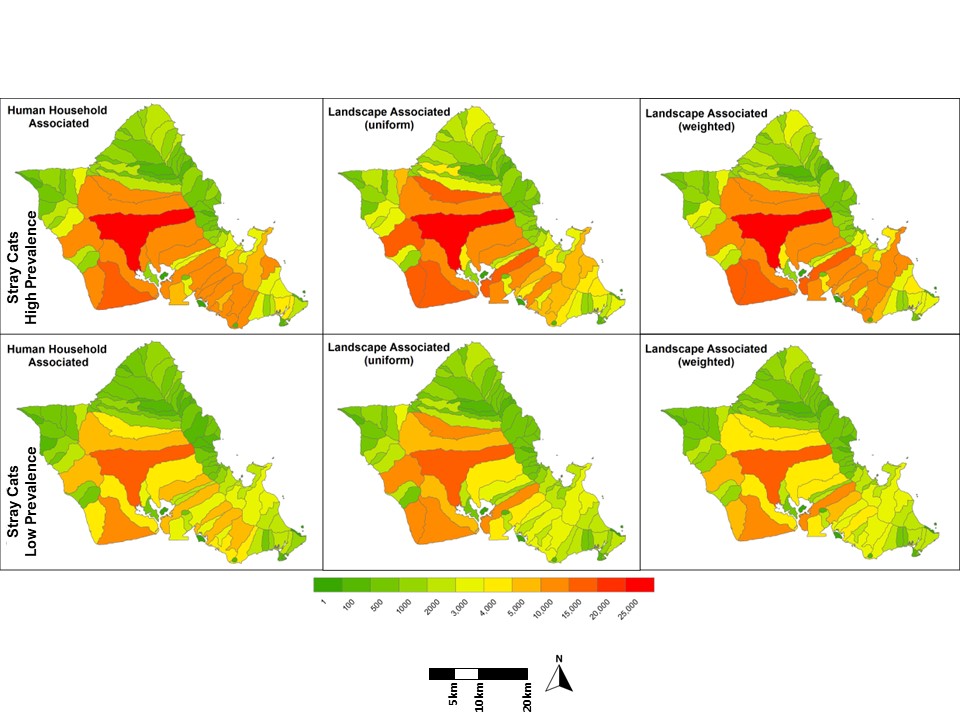


### Figure SI6: Oocyst export summarized by watershed for all landscape scenarios for Wildland Feral cats (medium abundance, high shedding prevalence, annual model).


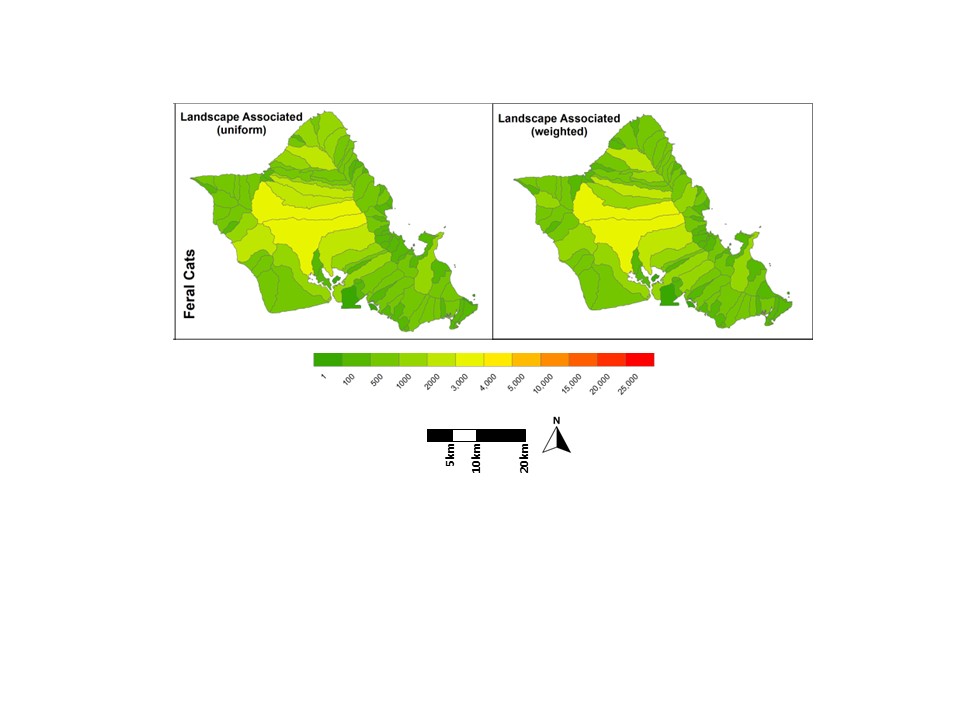


**Figure SI7: Hawaiian monk seal space use around O**'**ahu based on satellite telemetry of 91 seals from 2007 - 2019.** The color of each 1km cell represents the proportion of total seal space use occurring in that cell.


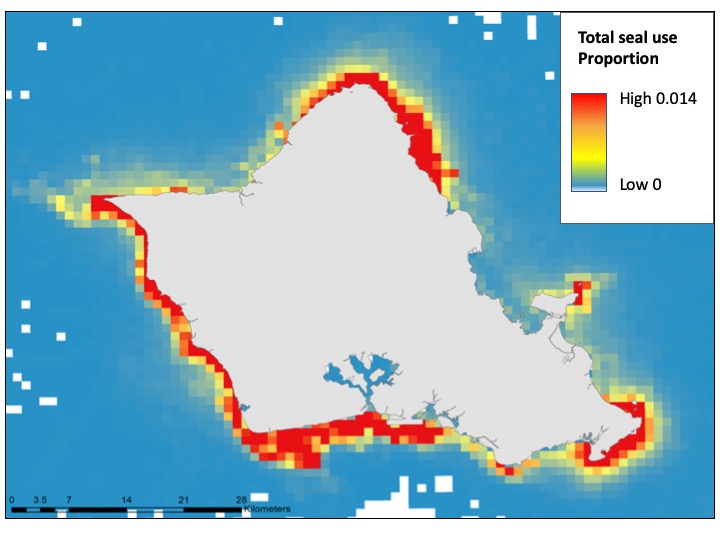


**Supplemental Model Application Overview**

Recognizing the diversity of management goals and potential actions, we wanted to provide managers with the tools to evaluate scenarios we did not cover in our model. In this paper we aimed to estimate the most realistic risk distribution currently impacting the island of O'ahu. However, we did not delve into all possible changes that could be affected through numerous management actions. We built this web-based application to allow managers to alter the number of cats, distribution of cats, and shedding rates so that they might evaluate outcomes of potential management options. Note, this model is not equipped to evaluate the effectiveness of particular management methods – i.e. we would not be able to answer whether method X is sufficient to decrease the cat population by Y percent. However, given the assumption that the manager has effectively decreased the cat population in some landscape by some percentage, then the model can then estimate the change in oocyst exposure risk that will result from that management outcome. The application is housed at NOAAs R Studio Connect server: <https://connect.fisheries.noaa.gov/content/d1069bd2-c505-42d8-bee5-b04cbad6e5ea>.

This app is designed to generate maps of oocyst contamination across Oahu. You will also have the option to use those oocyst contamination maps in further hydrological modeling to estimate oocyst export to coastal waters.

**INPUT Oocyst Contamination Tab –**

1: Estimate Cat Abundance

2: Estimate T. gondii Oocyst Shedding Prevalence

3: Estimate Landscape Distribution

4: Press the “Refresh Model Results” button to run the model with a new scenario.

**RESULTS: Oocyst Contamination Graphs Tab –**

In this tab you can view the maps of oocyst contamination for each cat type based on the parameters you selected in the INPUT tab. You will also see a bar graph comparing the total oocyst contamination load for each cat type, along with some basic summary statistics.

**DOWNLOADS: Tables Tab –**

You may choose to use this tab or not. If you are primarily interested in viewing oocyst contamination levels in the terrestrial environment – you won’t need these tables at all. Enjoy the maps on the RESULTS tab. However, if you want to use your oocyst contamination scenario to run a hydrological model to estimate oocyst export into coastal waters, you will need to take extra steps. This app generates input tables to run the InVEST NDR hydrological model. Download the tables from your scenario and submit them to Michelle.Barbieri@noaa.gov to run through the hydrological model.

**Supplemental References**

Cove MV, Gardner B, Simons TR, Kays R, O’Connell AF. Free-ranging domestic cats (Felis catus) on public lands: estimating density, activity, and diet in the Florida Keys. Biological Invasions. 2018;20(2):333-44.

Falinski K. Predicting sediment export into tropical coastal ecosystems to support ridge to reef management. Health. 2016;5:83-95.

Goltz DM, Hess SC, Brinck KW, Banko PC, Danner RM. Home Range and Movements of Feral Cats on Mauna Kea, Hawai? i. Pacific Conservation Biology. 2008;14(3):177-84.

Hamel P, Falinski K, Sharp R, Auerbach DA, Sánchez-Canales M, Dennedy-Frank PJ. Sediment delivery modeling in practice: Comparing the effects of watershed characteristics and data resolution across hydroclimatic regions. Science of the Total Environment. 2017;580:1381-8.

Hess SC, Banko PC, Hansen H. An adaptive strategy for reducing feral cat predation on endangered Hawaiian birds. Pacific Conservation Biology. 2009;15(1):56-64.

Kwong KNK, Bholah A, Volcy L, Pynee K. Nitrogen and phosphorus transport by surface runoff from a silty clay loam soil under sugarcane in the humid tropical environment of Mauritius. Agriculture, Ecosystems & Environment. 2002;91(1-3):147-57.

Lohr CA, Cox LJ, Lepczyk CA. Costs and benefits of trap‐neuter‐release and euthanasia for removal of urban cats in Oahu, Hawaii. Conservation Biology. 2013;27(1):64-73.

Sharp R, Talli sH, Ricketts T, Guerry A, Wood S, Chaplin-Kramer R, et al. InVEST 3.2 User's Guide. The Natural Capital Project Stanford University, University of Minnesota, The Nature Conservancy, and World Wildlife Fund. 2015.

US Census. American Community Survey 2015, accessed from <https://census.hawaii.gov/acs/acs-2015/>. 2015.

Ward Research Inc. Executive Summary of Cat Ownship and Colony Care Survey. present by Hawaiin Humane Society at The Outdoor Cat Symposium. 2012.

Ward Research Inc. Public Attitudes Toward the Hawaiian Humane Society and Animal Related Issues. A contracted report prepared for Hawaiian Humane Society. 2018.
